# Design, Synthesis and Biological Evaluation of Hydroxamic Acid Derivatives as Potential High Density Lipoprotein (HDL) Receptor CLA-1 Up-Regulating Agents

**DOI:** 10.3390/molecules16119178

**Published:** 2011-11-02

**Authors:** Xiaofang Chen, Li Wang, Yu Du, Yanbin Wu, Xiaojian Jia, Yuan Yang, Bin Hong

**Affiliations:** Institute of Medicinal Biotechnology, Chinese Academy of Medical Sciences & Peking Union Medical College, Beijing 100050, China; Email: capricornkey@163.com (L.W.); duyu1983@hotmail.com (Y.D.); wyblily@sina.com (Y.W.); xiaojianjia@yahoo.cn (X.J.); yangyuan78@hotmail.com (Y.Y.)

**Keywords:** high density lipoprotein receptor, up-regulator, CLA-1, trichostatin A (TSA), suberoylanilide hydroxamic acid (SAHA), hydroxamic acid

## Abstract

Trichostatin A (TSA) and suberoylanilide hydroxamic acid (SAHA) were reported in our recent publication as novel human high density lipoprotein (HDL) receptor CD36 and Lysosomal integral membrane protein-II Analogous-1 (CLA-1) up-regulators. As part of a broader effort to more fully explore the structure-activity relationships (SAR) of CLA-1 up-regulators, we synthesized a series of hydroxamic acid derivatives and evaluated their CLA-1 up-regulating activities in HepG2 cells. Some compounds exhibited over 10-fold up-regulation of CLA-1 expression in HepG2 cells at 10 μg/mL concentration. The compound **1g** showed the best potency, with a lower EC_50_ than TSA (EC_50_ = 0.32 μM *versus* 1.2 μM). These compounds provide early new CLA-1 up-regulators with potential for treating atherosclerosis.

## 1. Introduction

Atherosclerosis is one of the leading causes of mortality in industrialized and developing nations. It is a progressive disease that is characterized by the accumulation of lipid-rich plaques within the walls of arteries [1]. Long-term clinical studies have shown that plasma concentrations of high density lipoprotein (HDL) cholesterol (HDL-C) are inversely proportional to the risk for atherosclerotic cardiovascular disease. One of the major atheroprotective actions of HDL particles involves the transport of excess cholesterol from peripheral tissues to the liver for excretion, a process known as reverse cholesterol transport (RCT) [2]. HDL-mediated RCT represents a major target for the development of innovative antiatherogenic strategies to reduce the risk of atherosclerotic cardiovascular disease.

Scavenger receptor class B type I (SR-BI) is the first molecularly well-defined HDL receptor in mice [3], and its human homologue is CD36 and Lysosomal integral membrane protein-II Analogous-1 (CLA-1) [4]. SR-BI/CLA-1 plays an important role in RCT by mediating selective uptake of cholesteryl ester from peripheral tissues to the liver. The discovery of up-regulators of CLA-1 expression may benefit the further study of the mechanism of action of CLA-1 in human atherosclerotic cardiovascular diseases and might have pharmacologic applications [5].

**Figure 1:**
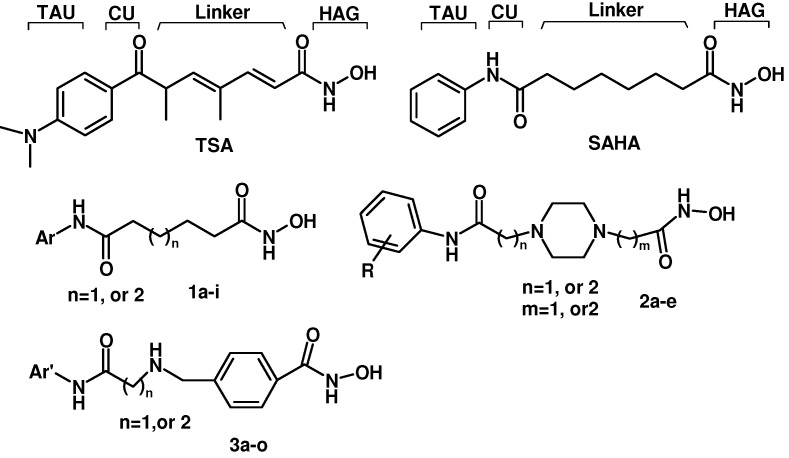
Structures of TSA, SAHA and designed compounds.

To obtain active compounds that can increase CLA-1 transcriptional level in liver cells, we developed a cell-based reporter assay applicable for high-throughput screening (HTS) [6]. Using this assay trichostatin A (TSA) was found to prominently up-regulate CLA-1 transcriptional activity [7]. Suberoylanilide hydroxamic acid (SAHA), an analogue of TSA, was also found to up-regulate CLA-1 transcriptional activity [7]. TSA and SAHA have some common pharmacophore characteristics, which can be segmented into four parts: (i) a terminal aromatic unit (TAU); (ii) a connecting unit (CU); (iii) a linker domain; and (iv) the hydroxamic acid group (HAG) (Figure 1) [8,9]. Small molecular structure changes have been found to have significant impact on up-regulating activity. When the hydroxamic acid group was replaced by a carboxyl and an acylamide, the two corresponding analogues of TSA showed a 10-fold and a 250-fold decrease in maximal up-regulating fold compared with TSA, respectively [10]. To better understand the SAR of hydroxamic acid compounds, to lay out a foundation for potential up-regulators of CLA-1 expression, and to further explore the mechanism of CLA-1/SR-BI promoter up-regulation we embarked on the design and synthesis of analogues library of TSA and SAHA. 

Based on TSA and SAHA, primarily the hydroxamic acid group was retained and the lead change program focused on the linker and terminal aromatic units. In TSA the chain length between the CU and the hydroxamic acid group is five atoms and it is six atoms for SAHA. Firstly we adjusted the length of the linker to 4–5 carbon atoms based on SAHA to obtain compounds **1a-i**. TSA has two methyl groups on the linker part, so piperazine ring was introduced in different locations in the linker in **2a-e**, mainly based on SAHA, to investigate space tolerance. Furthermore we introduced a benzylamino group to the linker part to synthesize compounds **3a-o** to investigate its effect on up-regulating activity. Compounds **12a-d** with a carboxyl group replacing the hydroxamic acid group of **3a-d** were also investigated.

### 1.1. Chemistry

**Scheme 1:**
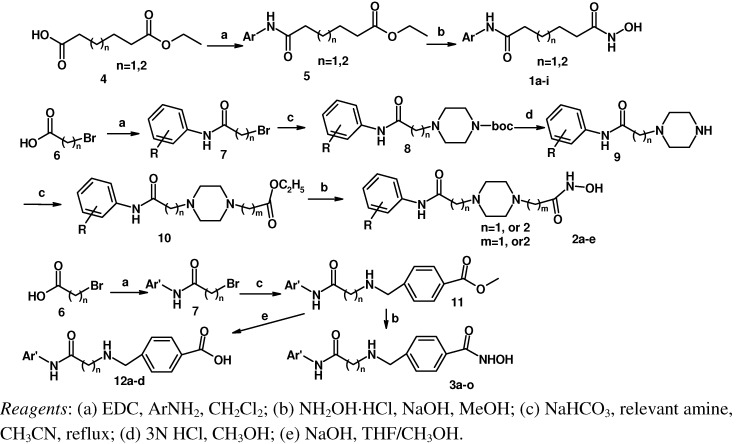
The synthesis route of objective compounds.

Scheme 1 shows the synthetic routes used to prepare the three series of hydroxamic acid compounds **1a-i**, **2a-e** and **3a-o**. Reactions of adipic acid monoethyl ester or pimelic acid monoethyl ester **4** with relevant amines using 1-ethyl-3-(3-dimethyllaminopropyl)carbodiimide hydrochloride (EDC·HCl) as a coupling agent gave acylamides **5** [11], which were then reacted with hydroxylamine in the presence of NaOH, yielding the desired targets **1a-i** [12]. Bromoacetic acid or 3-bromopropionic acid **6** were reacted with relevant amines using EDC·HCl to obtain acylamides **7**, which were in turn reacted with 1-BOC-piperazine in the presence of K_2_CO_3_ to give **8**. The BOC groups of compounds **8** were deprotected with 3 N HCl to afford **9** [13], which were reacted with ethyl bromoacetate or ethyl 3-bromopropionate in the presence of K_2_CO_3_ to give **10** [14]. Compounds **10** were finally converted into **2a-e** in the same way as described for **1a-i**. Acylamides **7** with methyl 4-(aminomethyl)benzoate hydrochloride in the presence of KHCO_3_ furnished **11**, which were then converted to **3a-o** according to the synthetic method used for **1a-i**.

## 2. Results and Discussion

As exploratory screening of the synthesized compounds, we first evaluated the up-regulating of CLA-1 expression activities in HepG2 cells at 10 μg/mL concentration. Table 1, Table 2, Table 3 summarize the activity data and inhibition rates of HDAC at 500 nM for the synthesized compounds as well as the known up-regulators TSA and SAHA as positive controls.

**Table 1:** Up-regulating activities of CLA-1 expression in HepG2 cells and inhibition rate of HDAC of compounds **1a-i**. 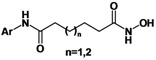

Cpd. ^a^	Ar	n	Up-regulating fold ^b^	Inhibition rate
			(SD ^c^)	of HDAC (%) ^d^
**1a**	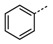	**1**	**5.4** **(1.4)**	**8.8**
**1b**	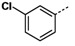	**1**	**9.4** **(1.3)**	**18.8**
**1c**	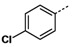	**1**	**5.9** **(1.8)**	**18.8**
**1d**	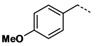	**1**	**3.6** **(0.3)**	**9.6**
**1e**	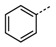	**2**	**13.8** **(2.1)**	**34.9**
**1f**	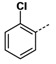	**2**	**9.2** **(2.3)**	**23.2**
**1g**	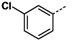	**2**	**13.3** **(3.4)**	**92.1**
**1h**	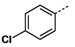	**2**	**10.4** **(1.4)**	**51.2**
**1i**	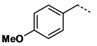	**2**	**15.7** **(2.5)**	**39.9**
**SAHA**			**15.5** **(0.5)**	**72.9**
**TSA**			**35.7** **(1.8)**	**95.0**

^a^ All compounds tested were >95% pure by HPLC; ^b^ All compounds were tested at 10 μg/mL except that SAHA was at 2.5 μM and TSA at 3.0 μM; ^c^ Standard deviation; ^d^ All compounds were tested at 500 nM.

**Table 2:** Up-regulating activities of CLA-1 expression in HepG2 cells and inhibition rate of HDAC of compounds **2a-e**. 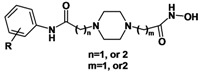

Cpd.	R	n	m	Up-regulating fold	Inhibition rate
				(SD)	of HDAC (%)
**2a**	**H**	**2**	**1**	**1.2** **(0.1)**	**5.4**
**2b**	**2,4-(OCH3)**	**2**	**1**	**1.1** **(0.08)**	**7.2**
**2c**	**H**	**1**	**1**	**1.1** **(0.1)**	**5.6**
**2d**	**4-N(CH3)2**	**1**	**1**	**1.1** **(0.2)**	**5.9**
**2e**	**H**	**1**	**2**	**1.1** **(0.1)**	**8.6**

**Table 3:** Up-regulating activities of CLA-1 expression in HepG2 cells and inhibition rate of HDAC of compounds **3a-o**, **12a-d**. 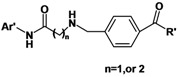

Cpd.	Ar’	n	R’	Up-regulating fold	Inhibition rate
				(SD)	of HDAC (%)
**3a**	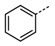	**1**	**NHOH**	**10.1** **(0.8)**	**23.3**
**3b**	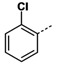	**1**	**NHOH**	**6.6** **(0.02)**	**23.7**
**3c**	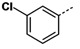	**1**	**NHOH**	**0.89** **(0.05)**	**34.2**
**3d**	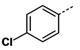	**1**	**NHOH**	**12.4** **(1.1)**	**28.0**
**3e**	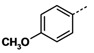	**1**	**NHOH**	**10.5** **(0.6)**	**28.3**
**3f**	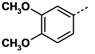	**1**	**NHOH**	**2.9** **(0.4)**	**21.3**
**3g**	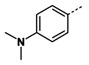	**1**	**NHOH**	**8.1** **(0.3)**	**9.8**
**3h**	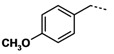	**1**	**NHOH**	**4.6** **(0.1)**	**29.8**
**3i**	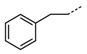	**1**	**NHOH**	**5.0** **(0.4)**	**16.5**
**3j**	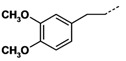	**1**	**NHOH**	**1.9** **(0.4)**	**10.9**
**3k**	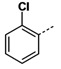	**2**	**NHOH**	**8.2** **(1.6)**	**35.0**
**3l **	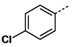	**2**	**NHOH**	**13.4** **(4.8)**	**45.7**
**3m**	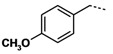	**2**	**NHOH**	**5.2** **(1.1)**	**25.5**
**3n**	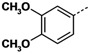	**2**	**NHOH**	**1.4** **(0.3)**	**13.0**
**3o**	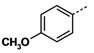	**2**	**NHOH**	**7.2** **(1.8)**	**27.7**
**12a**	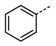	**1**	**COOH**	**1.5** **(0.3)**	**-**
**12b**	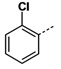	**1**	**COOH**	**1.1** **(0.02)**	**-**
**12c**	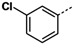	**1**	**COOH**	**1.1** **(0.1)**	**-**
**12d**	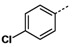	**1**	**COOH**	**1.2** **(0.1)**	**-**

**Figure 2:**
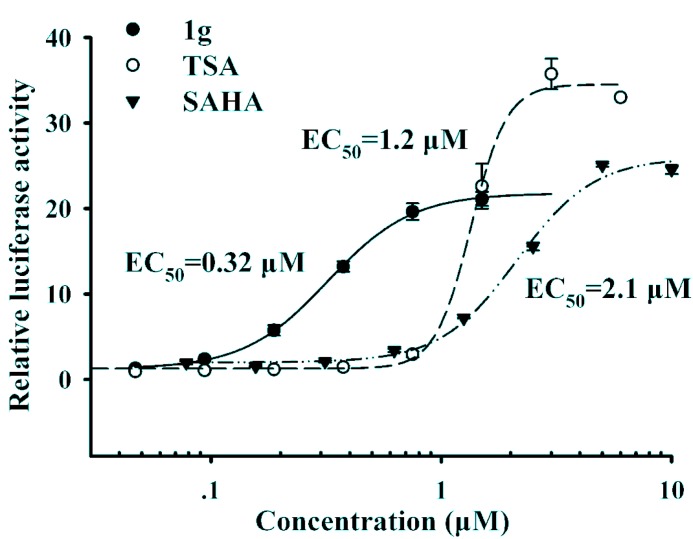
The dose-response curves of compound **1g** and SAHA.

Compounds **1a-i** with a linker chain of four or five carbon atoms showed promising up-regulating activity. In general, compounds **1e-i** with five carbon atoms chain showed more powerful activity than **1a-d** with 9.2~15.7 fold up-regulation. From the structural perspective, the carbon chain length has great influence on up-regulating activity; the longer chain with five carbon atoms is more active than the shorter one. Compounds **1a-e**, **1g**, **1i** were selected for further studies to obtain their activity EC_50_ values. Similarly compounds with five carbon atoms chain showed better activities than those with four carbon atoms chain. Figure 2 shows that compound **1g** with a 3-chloro substituent on the phenyl ring exhibited the best up-regulating activity in terms of EC_50_, suggesting that the substitution of the phenyl ring seemed to have significant impact on the up-regulating activity. Compound **1g** has an EC_50_ value of 0.32 μM, which is much lower than those of the reference compounds TSA (EC_50_ = 1.2 μM) and SAHA (EC_50_ = 2.1 μM), and more importantly, it showed much better up-regulating activity than both reference compounds at low concentrations (at and below 1 μM).

In contrast, **2a-e** incorporating a piperazine group on the linker part exhibited no up-regulating activity, suggesting that the position 1 to 2 carbon atoms away from hydroxamic acid can’t tolerate the bulky piperazine ring group.

**Figure 3:**
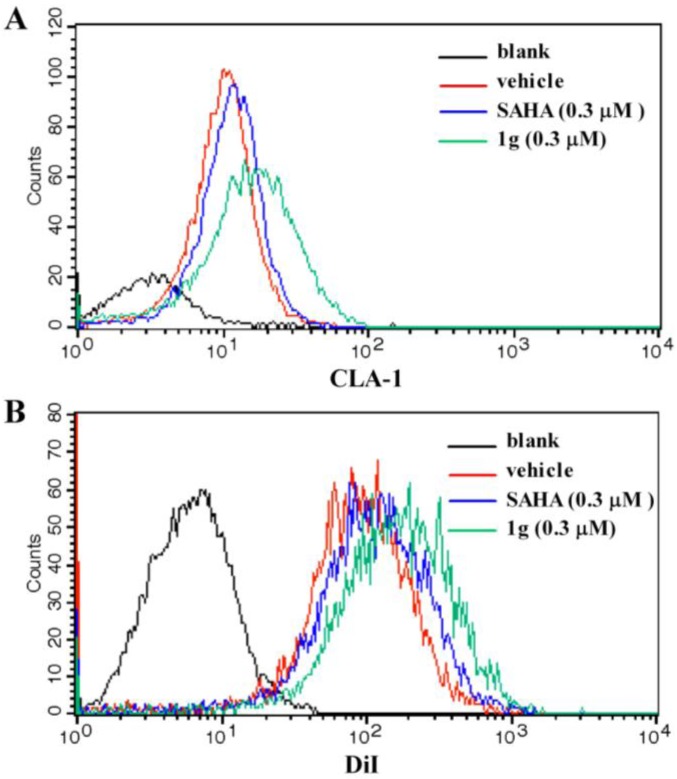
Effect of compound **1g** on CLA-1 expression and DiI-HDL uptake.

To determine if the CLA-1 expression level was increased by **1g** due to the up-regulation of transcriptional activity, flow cytometry was performed to investigate the abundance of CLA-1 protein levels in HepG2 cells with and without **1g** treatment. The result showed that with treatment of 0.3 μM **1g**, the protein level of CLA-1 was increased by 224.6% (Figure 3A), which was higher than 0.3 μM SAHA (63.5%). To test whether **1g** enhanced the selective uptake of lipids from HDL by increasing the expression of CLA-1, fluorescence-labeled DiI-HDL uptake after 12 h incubation with HepG2 cells was measured in the presence or absence of **1g**. 0.3 μM **1g** promoted the uptake of DiI-HDL into the HepG2 cells by 62.5% which was higher than 0.3 μM SAHA (24.1%) (Figure 3B).

**Figure 4:**
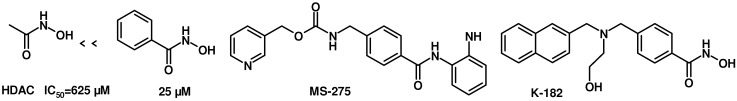
Structures of HDAC inhibitors.

TSA was reported as a potent specific reversible inhibitor of mammalian histone deacetylase (HDAC), leading to hyperacetylation of chromatin-associated histones and thereby open promoter loci to permit interaction with transcription factors to promote gene expression [15]. SAHA was the first HDAC inhibitor approved for treating cutaneous T cell lymphoma by FDA in 2006. It was reported that there was a 30-fold increase in HDAC inhibitory activity progressing from aceto- to benzoyl hydroxamic acid, with IC_50_ values of 625 and 25 μM, respectively (Figure 4) [16,17]. In addition, **MS-275** is under clinical trials and **K-182** is powerful as HDAC inhibitors [18]. They all possess a benzylamino group on the linker part. We also tried to introduce a benzylamino moiety into the linker part to synthesize compounds **3a-o**. Compounds **3a-o** showed moderate activity. When n = 1, phenyl **3a**, *p*-chloro phenyl **3d**, *p*-methoxyphenyl **3e** showed activity, with over 10-fold up-regulation at 10 μg/mL concentration, while **3b** and **3c** with chloro group substitution in the *ortho* and *meta* positions show a great decline in activity. Likewise, 3,4-dimethoxyphenyl compound **3f** also displayed reduced activity. Benzyl or phenylethyl compounds **3h-3j** were inferior to those with phenyl groups. When n = 2, *p*-chlorophenyl derivative **3l** was more potent than the *o*-chlorophenyl one **3k**. The 3,4-dimethoxy-phenyl compound **3n** showed a fourfold decrease in activity compared with the similar *p*-methoxy-phenyl analog **3m**.

For **12a-d**, the most notable observation is the lack of activity of all the carboxylic acid derivatives tested; conversely, nearly all hydroxamic acid analogs showed promising up-regulating activity. Consistent with our previous results [10], these results indicated that the hydroxamic acid group is indispensable. For this series of compounds, substitution at the *para*-position of the phenyl ring is preferred for good activity.

At present, there are only some clues that several known HDAC inhibitors such as TSA, SAHA and sodium butyrate are active on CLA-1 up-regulating activity [7]. However, TSA up-regulated CLA-1 transcription with EC_50_ = 1.2 μM, which is much higher than its HDAC inhibition IC_50_ values (usually at nanomolar level). As the relationship between HDAC inhibition and CLA-1 transcriptional upregulation is quite intriguing, we further detected the HDAC inhibitory activity of our compounds using a HDAC Fluorimetric Assay kit (Enzo Life Science). At 10 nM, none of all 29 compounds showed HDAC inhibition (<5% inhibition) except compound **1g** (~25% inhibition), whereas TSA exhibited ~60% inhibition at the same concentration (data not shown). At 500 nM, **1a-i** and **3a-o** showed different HDAC inhibition rates ranging from 8.8% to 92.1% and compounds **2a-e** showed little inhibition (<8.6%) (Table 1, Table 2, Table 3). Compound **1g** showed the highest HDAC inhibition rate of 92.1% whereas TSA and SAHA exhibited 72.9% and 95.0% inhibition, respectively (Table 1, Table 2, Table 3). The HDAC inhibition activity of the compounds synthesized in the present study correlated positively to their CLA-1 up-regulation activity on the whole, suggesting CLA-1 up-regulation may be dependent on the HDAC inhibition. However, the activity of some compounds towards HDAC inhibition did not correlate with the degree of up-regulation of CLA-1 promoter. This suggests that some of the hydroxamic acid derivatives may affect CLA-1 transcription through mechanisms other than HDAC inhibition in HepG2 cells. It will be interesting to determine the detailed relationship between HDAC inhibition and the induction of CLA-1 transcription in future studies.

## 3. Experimental

### 3.1. General

All reagents and solvents were reagent grade or were purified by standard methods before use. Melting points were determined in open capillaries on a RT-1 melting point apparatus (Tianjin Fenxi Yiqichang, Tianjin, China) and are uncorrected. Column chromatography was carried out on flash MCI GEL 20Y. ^1^H-NMR spectra analysis was performed on a Varian Inova 400 MHz spectrometer (Varian, Palo Alto, CA, USA), using DMSO-d_6_ as solvent and Me_4_Si as the internal standard. Chemical shifts (δ values) and coupling constants (J values) are given in ppm and Hz, respectively. ESI high-resolution mass spectra (HRMS) analysis was recorded on an Autospec Ultima-TOF mass spectrometer (Micromass UK Ltd., Manchester, UK). All the HRMS data were within ±5 ppm of calculated values.

### 3.2. General Procedure for the Amide Bond Formation (Scheme 1, step a)

A solution of aniline (1.86 g, 20.0 mmol) in CH_2_Cl_2_ (10.0 mL) was added dropwise to a cooled (−5 °C) solution of 3-bromopropionic acid (3.06 g, 20.0 mmol) and EDC·HCl (3.84 g, 20.0 mmol) in CH_2_Cl_2_ (20.0 mL). The mixture was allowed to warm at room temperature and stirred overnight. The progress of the reaction was monitored by Thin-Layer Chromatography (TLC; eluent: dichloromethane/ethyl acetate 5:1). After disappearance of the starting material, the mixture was washed with 1 mol/L HCl (30 mL), 5% solution of Na_2_HCO_3_ (30 mL) and brine. The organic layer was dried over anhydrous sodium sulfate and concentrated to give the crude material (about 60% yield) which was used for the next reaction.

### 3.3. General Procedure for the Amine Bond Formation (Scheme 1, step c)

The corresponding bromide (10.0 mmol), methyl 4-(aminomethyl)benzoate hydrochloride (2.02 g, 10.0 mmol) and potassium bicarbonate (1.50 g, 15.0 mmol) were dissolved in CH_3_CN (100 mL). The solution was stirred at reflux for 3–4 h, and then concentrated under reduced pressure. To the residue was added water and ethyl acetate. HCl solution (1 mol/L) was added to organic layer and precipitate was filtered. The sediment was dried (about 50% yield) and used for the next reaction.

### 3.4. General Procedure for Boc Deblocking (Scheme 1, step d)

Concentrated HCl (15 mL) was dropped into a solution of the corresponding BOC protected compound (5.82 mmol) in ethyl alcohol (50 mL), and the mixture was stirred for 16 h at room temperature. Saturated solution of NaHCO_3_ was added to pH > 8. The solution was concentrated under reduced pressure and the residue was extracted with ethyl acetate. The organic layer was concentrated under reduced pressure and provided the desired product as a yellow oil (about 80% yield).

### 3.5. General Procedure for the Synthesis of Hydroxamic Acids from Methyl Esters (Scheme 1, step b)

The corresponding methyl or ethyl esters **5**, **10**, **11** (5.0 mmol), hydroxylamine hydrochloride (280 mg, 4 mmol) were dissolved in MeOH (50 mL), and then 8 mol/L NaOH solution (10 mL) was added. The solution was stirred at ambient temperature for 1~2 h. To the solution was added 1 mol/L HCl (50 mL) at 0 °C until pH = 7~8, then the solution was concentrated under reduced pressure. The residue was purified by flash chromatography MCI GEL 20Y (water/methanol) to get the target compounds **1a-i**, **2a-e**, **3a-o**.

*Adipoyl anilide hydroxamic acid* (**1a**). A yellowish solid, 670 mg, yield: 59.2%; m.p. 180–181.5 °C; ^1^H-NMR: 1.52 (4H, m, 2CH_2_), 1.96 (2H, t, *J* = 6.8, CH_2_), 2.28 (2H, t, *J* = 6.8, CH_2_), 7.00 (1H, t, *J* = 8.0, Ar-H), 7.27 (2H, t, *J* = 8.0, Ar-H), 7.55 (2H, d, *J* = 8.0, Ar-H), 8.65 (1H, s, CONHOH), 9.84 (1H, s, NHCO), 10.34 (1H, s, CONHOH); MS (ESI) *m/z*: 237 (M+H)^+^; HRMS (ESI) *m/z*: (M+H)^+^ calcd. for C_12_H_17_N_2_O_3_, 237.1234; found, 237.1227.

*Adipoyl (3-chlorophenyl)amide hydroxamic acid* (**1b**). A khaki solid, 712 mg, yield: 67.4%; m.p. 169–171 °C; ^1^H-NMR: 1.52 (4H, m, 2CH_2_), 1.96 (2H, t, *J* = 6.8, CH_2_), 2.29 (2H, t, *J* = 6.8, CH_2_), 7.07 (1H, d, *J* = 8.0, Ar-H), 7.30 (1H, t, *J* = 8.0, Ar-H), 7.42 (1H, d, *J* = 8.0, Ar-H), 7.81 (1H, s, Ae-H), 8.65 (1H, s, CONHOH), 10.05 (1H, s, NHCO), 10.34 (1H, s, CONHOH). MS (ESI) m/z: 271 (M+H)^+^; HRMS (ESI) *m/z*: (M+H)^+^ calcd. for C_12_H_16_ClN_2_O_3_, 271.0849; found, 271.0839.

*Adipoyl (4-chlorophenyl)amide hydroxamic acid* (**1c**). A white solid, 543 mg, yield: 71.0%; m.p. 176–177 °C; ^1^H-NMR: 1.52 (4H, m, 2CH_2_), 1.96 (2H, t, *J* = 6.8, CH_2_), 2.28 (2H, t, *J* = 6.8, CH_2_), 7.32 (2H, d, *J* = 8.8, Ar-H), 7.60 (2H, d, *J* = 8.8, Ar-H), 8.65 (1H, s, CONHOH), 9.99 (1H, s, NHCO), 10.33 (1H, s, CONHOH); MS (ESI) *m/z*: 271 (M+H)^+^; HRMS (ESI) *m/z*: (M+H)^+^ calcd. for C_12_H_16_ClN_2_O_3_, 271.0849; found, 271.0836.

*Adipoyl (4-methoxybenzyl)amide hydroxamic acid* (**1d**). A khaki solid, 398 mg, yield: 35.9%; m.p. 140–142 °C; ^1^H-NMR: 1.46 (4H, m, 2CH_2_), 1.93 (2H, t, *J* = 6.8, CH_2_), 2.09 (2H, t, *J* = 6.8, CH_2_), 3.71 (3H, s, OCH_3_), 4.16 (2H, d, *J* = 6.0, CH_2_NH), 6.86 (2H, d, *J* = 8.4, Ar-H), 7.14 (2H, d, *J* = 8.4, Ar-H), 8.21 (1H, t, *J* = 6.0, NHCO), 8.65 (1H, s, CONHOH), 10.34 (1H, s, CONHOH); MS (ESI) *m/z*: 281 (M+H)^+^; HRMS (ESI) *m/z*: (M+H)^+^ calcd. for C_14_H_21_N_2_O_4_, 281.1496; found, 281.1487.

*Pimeloyl anilide hydroxamic acid* (**1e**). A khaki solid, 666 mg, yield: 75.8%; m.p. 148–150 °C; ^1^H-NMR: 1.26 (2H, m, CH_2_), 1.53 (4H, m, 2CH_2_), 1.93 (2H, t, *J* = 7.2, CH_2_), 2.27 (2H, t, *J* = 7.2, CH_2_), 7.00 (1H, t, *J* = 8.0, Ar-H), 7.26 (2H, t, *J* = 8.0, Ar-H), 7.57 (2H, d, *J* = 8.0, Ar-H), 8.64 (1H, s, CONHOH), 9.83 (1H, s, NHCO), 10.31 (1H, s, CONHOH); MS (ESI) *m/z*: 251 (M+H)^+^; HRMS (ESI) *m/z*: (M+H)^+^ calcd. for C_13_H_18_N_2_O_3_, 251.1390; found, 251.1385.

*Pimeloyl (2-chlorophenyl)amide hydroxamic acid* (**1f**). A white solid, 359 mg, yield: 43.8%; m.p. 62–64 °C; ^1^H-NMR: 1.28 (2H, m, CH_2_), 1.47 (4H, m, 2CH_2_), 1.92 (2H, m, CH_2_), 2.33 (2H, m, CH_2_), 7.17 (1H, m, Ar-H), 7.30 (1H, t, *J* = 7.2, Ar-H), 7.47 (1H, m, Ar-H), 7.65 (1H, d, *J* = 7.2, Ar-H), 8.64 (1H, s, CONHOH), 9.43 (1H, s, NHCO), 10.32 (1H, s, CONHOH); MS (ESI) *m/z*: 285 (M+H)^+^; HRMS (ESI) *m/z*: (M+H)^+^ calcd. for C_13_H_18_ClN_2_O_3_, 285.1001; found, 285.0995.

*Pimeloyl (3-chlorophenyl)amide hydroxamic acid* (**1g**). A white solid, 723 mg, yield: 77.2%; m.p. 138–140 °C; ^1^H-NMR: 1.25 (2H, m, CH_2_), 1.53 (4H, m, 2CH_2_), 1.93 (2H, t, *J* = 7.2, CH_2_), 2.29 (2H, t, *J* = 7.2, CH_2_), 7.06 (1H, dd, *J* = 8.0, 2.0, Ar-H), 7.30 (1H, t, *J* = 8.0, Ar-H), 7.42 (1H, d, *J* = 8.0, Ar-H), 7.81 (1H, t, *J* = 2.0, Ar-H), 8.64 (1H, s, CONHOH), 10.04 (1H, s, NHCO), 10.31 (1H, s, CONHOH); MS (ESI) *m/z*: 285 (M+H)^+^; HRMS (ESI) *m/z*: (M+H)^+^ calcd. for C_13_H_18_ClN_2_O_3_, 285.1001; found, 285.0994.

*Pimeloyl (4-chlorophenyl)amide hydroxamic acid* (**1h**). A white solid, 219 mg, yield: 53.3%; m.p. 161–162 °C; ^1^H-NMR: 1.25 (2H, m, CH_2_), 1.53 (4H, m, 2CH_2_), 1.93 (2H, t, *J* = 7.6, CH_2_), 2.28 (2H, t, *J* = 7.6, CH_2_), 7.32 (2H, d, *J* = 8.8, Ar-H), 7.60 (2H, d, *J* = 8.8, Ar-H), 8.63 (1H, s, CONHOH), 9.98 (1H, s, NHCO), 10.31 (1H, s, CONHOH); MS (ESI) *m/z*: 285 (M+H)^+^; HRMS (ESI) *m/z*: (M+H)^+^ calcd. for C_13_H_18_ClN_2_O_3_, 285.1001; found, 285.0995.

*Pimeloyl (4-methoxybenzyl)amide hydroxamic acid* (**1i**). A white solid, 441 mg, yield: 41.7%; m.p. 133–135 °C; ^1^H-NMR: 1.20 (2H, m, CH_2_), 1.47 (4H, m, 2CH_2_), 1.91 (2H, t, *J* = 7.2, CH_2_), 2.08 (2H, t, *J* = 7.2, CH_2_), 3.71 (3H, s, OCH_3_), 4.16 (2H, d, *J* = 5.6, CH_2_NH), 6.86 (2H, d, *J* = 8.8, Ar-H), 7.14 (2H, d, *J* = 8.8, Ar-H), 8.22 (1H, t, *J* = 5.6, NHCO); MS (ESI) *m/z*: 295 (M+H)^+^; HRMS (ESI) *m/z*: (M+H)^+^ calcd. for C_15_H_23_N_2_O_4_, 295.1657; found, 295.1645.

*N-Hydroxy-2-(4-phenylcarbamoylethyl-piperazin-1-yl)-acetamide* (**2a**). A white solid, 35 mg, yield: 62.4%; m.p. 75–77 °C. ^1^H-NMR: 2.42–2.61 (12H, m, 6CH_2_), 3.28 (2H, s, CH_2_), 7.01 (1H, t, *J* = 7.6, Ar-H), 7.26 (2H, m, Ar-H), 7.55 (2H, d, *J* = 8.0, Ar-H), 10.06 (1H, m, NHCO); MS (ESI) *m/z*: 307 (M+H)^+^; HRMS (ESI) *m/z*: (M+H)^+^ calcd. for C_15_H_23_N_4_O_3_, 307.1765; found, 307.1754.

*N-Hydroxy-2-{4-[(3,4-dimethoxyphenylcarbamoyl)-ethyl]piperazin-1-yl}acetamide* (**2b**). A yellowish solid, 21 mg, yield: 54.9%; m.p. 69–71 °C; ^1^H-NMR: 2.39–2.58 (12H, m, 6CH_2_), 3.28 (2H, s, CH_2_), 3.69 (3H, s, OCH_3_), 3.70 (3H, s, OCH_3_), 6.85 (1H, d, *J* = 8.8, Ar-H), 7.04 (1H, m, Ar-H), 7.27 (1H, d, *J* = 2.0, Ar-H), 9.94 (1H, m, NHCO); MS (ESI) *m/z*: 367 (M+H)^+^; HRMS (ESI) *m/z*: (M+H)^+^ calcd. for C_17_H_27_N_4_O_5_, 367.1976; found, 367.1966.

*N-Hydroxy-2-(4-phenylcarbamoylmethylpiperazin-1-yl)acetamide* (**2c**). A yellowish solid, 13 mg, yield: 79.8%; m.p. 56–58 °C; ^1^H-NMR: 2.41–2.73 (8H, m, 4CH_2_), 3.09 (2H, s, CH_2_), 3.10 (2H, s, CH_2_), 7.04 (1H, t, *J* = 7.6, Ar-H), 7.26 (2H, m, Ar-H), 7.55 (2H, d, *J* = 8.0, Ar-H), 9.64 (1H, s, NHCO); MS (ESI) *m/z*: 293 (M+H)^+^; HRMS (ESI) *m/z*: (M+H)^+^ calcd. for C_14_H_21_N_4_O_3_, 293.1608; found, 293.1598.

*N-Hydroxy-2-{4-[(4-dimethylaminophenylcarbamoyl)methyl]piperazin-1-yl}acetamide* (**2d**). A white solid, 33 mg, yield: 44.4%; m.p. 217–219 °C; ^1^H-NMR: 2.48 (4H, m, 2CH_2_), 2.83 (10H, m, 2CH_2_, 2CH_3_), 3.03 (2H, s, CH_2_), 3.30 (3H, s, OCH_3_), 6.67 (2H, d, *J* = 9.2, Ar-H), 7.39 (2H, d, *J* = 9.2, Ar-H), 9.33 (1H, s, NHCO); MS (ESI) *m/z*: 336 (M+H)^+^; HRMS (ESI) *m/z*: (M+H)^+^ calcd. for C_16_H_26_N_5_O_3_, 336.2030; found, 336.2018.

*N-Hydroxy-3-(4-phenylcarbamoylmethylpiperazin-1-yl)propionamide* (**2e**). A white solid, 29 mg, yield: 65.1%; m.p. 55–57 °C; ^1^H-NMR: 2.23 (2H, m, CH_2_), 2.51–2.64 (10H, m, 5CH_2_), 3.18 (2H, s, CH_2_), 7.06 (1H, t, *J* = 7.6, Ar-H), 7.31 (2H, m, Ar-H), 7.55 (2H, d, *J* = 7.6, Ar-H), 8.77 (1H, s, CONHOH), 9.75 (1H, s, NHCO), 10.46 (1H, s, CONHOH); MS (ESI) *m/z*: 307 (M+H)^+^; HRMS (ESI) *m/z*: (M+H)^+^ calcd. for C_15_H_23_N_4_O_3_, 307.1765; found, 307.1753.

*N-Hydroxy-4-{[(phenylcarbamoylmethyl)amino]methyl}benzamide* (**3a**). A white solid, 67 mg, yield: 47.7%; m.p. 124–126 °C. ^1^H-NMR: 3.26 (2H, s, CH_2_CO), 3.77 (2H, s, NHCH_2_), 7.01–7.71 (9H, m, Ar-H), 9.80 (1H, s, CONH); MS (ESI) *m/z*: 300 (M+H)^+^; HRMS (ESI) *m/z*: (M+H)^+^ calcd. for C_16_H_18_N_3_O_3_, 334.0953; found, 334.0952.

*N-Hydroxy-4-{[(2-chlorophenylcarbamoylmethyl)amino]methyl}benzamide* (**3b**). A white solid, 72 mg, yield: 57.0%; m.p. 160–162 °C; ^1^H-NMR: 3.31 (2H, s, CH_2_CO), 3.82 (2H, s, NHCH_2_), 7.12 (1H, m, Ar-H), 7.33 (1H, m, Ar-H), 7.45 (2H, d, *J* = 8.0, Ar-H), 7.50 (1H, m, Ar-H), 7.71 (2H, d, *J* = 8.0, Ar-H), 8.24 (1H, m, Ar-H), 8.98 (1H, s, CONHOH), 10.05 (1H, s, NHCO), 11.15 (1H, s, CONHOH); MS (ESI) *m/z*: 334 (M+H)^+^; HRMS (ESI) *m/z*: (M+H)^+^ calcd. for C_16_H_17_ClN_3_O_3_, 334.0953; found, 334.0952.

*N-Hydroxy-4-{[(3-chlorophenylcarbamoylmethyl)amino]methyl}benzamide* (**3c**). A yellowish solid, 45 mg, yield: 66.4%; m.p. 127–129 °C; ^1^H-NMR: 3.27 (2H, s, CH_2_CO), 3.76 (2H, s, NHCH_2_), 7.09 (1H, d, *J* = 8.0, Ar-H), 7.32 (1H, t, *J* = 8.0, Ar-H), 7.41 (2H, d, *J* = 8.0, Ar-H), 7.48 (1H, d, *J* = 8.0, Ar-H), 7.70 (2H, d, *J* = 8.0, Ar-H), 7.84 (1H, s, Ar-H), 9.99 (1H, s, NHCO); MS (ESI) *m/z*: 334 (M+H)^+^; HRMS (ESI) *m/z*: (M+H)^+^ calcd. for C_16_H_17_ClN_3_O_3_, 334.0953; found, 334.0957.

*N-Hydroxy-4-{[(4-chlorophenylcarbamoylmethyl)amino]methyl}benzamide* (**3d**). A white solid, 67 mg, yield: 31.9%; m.p. 162–163 °C; ^1^H-NMR: 3.26 (2H, s, CH_2_CO), 3.77 (2H, s, NHCH_2_), 7.34 (2H, d, *J* = 8.4, Ar-H), 7.42 (2H, d, *J* = 8.0, Ar-H), 7.64 (2H, d, *J* = 8.4, Ar-H), 7.70 (2H, d, *J* = 8.0, Ar-H), 8.97 (1H, s, CONHOH), 9.94 (1H, s, NHCO), 11.14 (1H, s, CONHOH); MS (ESI) *m/z*: 334 (M+H)^+^; HRMS (ESI) *m/z*: (M+H)^+^ calcd. for C_16_H_17_ClN_3_O_3_, 334.0953; found, 334.0953.

*N-Hydroxy-4-{[(4-methoxyphenylcarbamoylmethyl)amino]methyl}benzamide* (**3e**). A white solid, 38 mg, yield: 68.9%; m.p. 156–157 °C; ^1^H-NMR: 2.79 (1H, brs, NH), 3.22 (2H, s, CH_2_CO), 3.71 (3H, s, OCH_3_), 3.76 (2H, s, NHCH_2_), 6.86 (2H, d, *J* = 8.8, Ar-H), 7.42 (2H, d, *J* = 8.0, Ar-H), 7.50 (2H, d, *J* = 8.8, Ar-H), 7.70 (2H, d, *J* = 8.0, Ar-H), 8.96 (1H, s, CONHOH), 9.66 (1H, s, NHCO), 11.14 (1H, s, CONHOH); MS (ESI) *m/z*: 330 (M+H)^+^; HRMS (ESI) *m/z*: (M+H)^+^ calcd. for C_17_H_20_N_3_O_4_, 330.1448; found, 330.1448.

N-Hydroxy-4-{[(3,4-dimethoxyphenylcarbamoylmethyl)amino]methyl}benzamide *(**3f**).* A white solid, 35 mg, yield: 57.3%; m.p. 175–177 °C; ^1^H-NMR: 3.16 (2H, s, CH_2_CO), 3.70 (3H, s, OCH_3_), 3.71 (3H, s, OCH_3_), 3.78 (2H, s, NHCH_2_), 6.86 (1H, d, *J* = 8.8, Ar-H), 7.12 (1H, dd, *J* = 8.8, 2.0, Ar-H), 7.30 (1H, d, *J* = 2.0, Ar-H), 7.43 (2H, d, *J* = 8.0, Ar-H), 7.71 (2H, d, *J* = 8.0, Ar-H), 8.97 (1H, s, CONHOH), 9.65 (1H, s, NHCO), 11.15 (1H, s, CONHOH); MS (ESI) *m/z*: 360 (M+H)^+^; HRMS (ESI) *m/z*: (M+H)^+^ calcd. for C_18_H_22_N_3_O_5_, 360.1554; found, 360.1543.

*N-Hydroxy-4-{[(4-dimethylaminephenylcarbamoylmethyl)amino]methyl}benzamide* (**3g**). A white solid, 17 mg, yield: 21.0%; m.p. 116–118 °C; ^1^H-NMR: 2.83 (6H, s, 2NCH_3_), 3.20 (2H, s, NHCH_2_), 3.76 (2H, s, NHCH_2_), 6.67 (2H, d, *J* = 9.2, Ar-H), 7.41 (4H, m, Ar-H), 7.70 (2H, d, *J* = 8.0, Ar-H), 8.97 (1H, s, NHCO), 9.50 (1H, s, CONHOH), 11.19 (1H, s, CONHOH); MS (ESI) *m/z*: 343 (M+H)^+^; HRMS (ESI) *m/z*: (M+H)^+^ calcd. for C_18_H_23_N_4_O_3_, 343.1765; found, 343.1777.

*N-Hydroxy-4-{[(4-methoxybenzylcarbamoylmethyl)amino]methyl}benzamide* (**3h**). A white solid, 31 mg, yield: 60.2%; m.p. 71–73 °C; ^1^H-NMR: 3.09 (2H, s, CH_2_CO), 3.69 (2H, s, NHCH_2_), 3.71 (3H, s, OCH_3_), 4.22 (2H, d, *J* = 6.0, CH_2_), 6.86 (2H, d, *J* = 8.4, Ar-H), 7.16 (2H, d, *J* = 8.4, Ar-H), 7.38 (2H, d, *J* = 8.4, Ar-H), 7.68 (2H, d, *J* = 8.4, Ar-H), 8.22 (1H, t, *J* = 6.0, NHCO), 8.96 (1H, s, CONHOH), 11.14 (1H, s, CONHOH); MS (ESI) *m/z*: 344 (M+H)^+^; HRMS (ESI) *m/z*: (M+H)^+^ calcd. for C_18_H_22_N_3_O_4_, 344.1605; found, 344.1605.

*N-Hydroxy-4-{[(phenethylcarbamoylmethyl)amino]methyl}benzamide* (**3i**). A yellow solid, 53 mg, yield: 57.7%; m.p. 114–116 °C; ^1^H-NMR: 2.72 (2H, t, *J* = 7.2, NHCH_2_CH_2_), 3.02 (2H, s, CH_2_CO), 3.31 (2H, t, *J* = 7.2, CH_2_CH_2_Ph), 3.62 (2H, s, NHCH_2_), 7.15–7.40 (7H, m, Ar-H), 7.68 (2H, d, *J* = 8.0, Ar-H), 7.84 (1H, m, NH), 8.97 (1H, s, CONHOH), 11.15 (1H, s, CONHOH); MS (ESI) *m/z*: 328 (M+H)^+^; HRMS (ESI) *m/z*: (M+H)^+^ calcd. for C_18_H_22_N_3_O_3_, 328.1656; found, 328.1651.

*N-Hydroxy-4-{[(3,4-dimethoxyphenethylcarbamoylmethyl)amino]methyl}benzamide* (**3j**). A white solid, 21 mg, yield: 37.9%; m.p. 123–125 °C. ^1^H-NMR: 2.65 (2H, t, *J* = 7.2, NHCH_2_), 3.02 (2H, s, CH_2_CO), 3.30 (2H, t, *J* = 7.2, ArCH_2_), 3.61 (2H, s, NHCH_2_), 3.69 (3H, s, OCH_3_), 3.71 (3H, s, OCH_3_), 6.69 (1H, m, Ar-H), 6.80 (2H, m, Ar-H), 7.31 (1H, d, *J* = 2.0, Ar-H), 7.80 (1H, t, *J* = 5.6, NHCO); MS (ESI) *m/z*: 388 (M+H)^+^; HRMS (ESI) *m/z*: (M+H)^+^ calcd. for C_20_H_26_N_3_O_5_, 388.1867; found, 388.1856.

*N-Hydroxy-4-{[(2-chlorophenylcarbamoylethyl)amino]methyl}benzamide* (**3k**). A white solid, 29 mg, yield: 71.5%; m.p. 57–59 °C; ^1^H-NMR: 2.48 (2H, t, *J* = 6.0, COCH_2_CH_2_), 2.79 (2H, t, *J* = 6.0, CH_2_CH_2_NH), 3.80 (2H, s, NHCH_2_), 7.11 (1H, t, *J* = 7.6, Ar-H), 7.29 (1H, t, *J* = 7.6, Ar-H), 7.41 (2H, d, *J* = 8.0, Ar-H), 7.45 (2H, d, *J* = 8.0, Ar-H), 7.69 (2H, d, *J* = 8.0, Ar-H), 7.98 (2H, d, *J* = 8.0, Ar-H), 8.96 (1H, s, CONHOH), 10.51 (1H, s, CONH), 11.14 (1H, s, CONHOH); MS (ESI) *m/z*: 348 (M+H)^+^; HRMS (ESI) *m/z*: (M+H)^+^ calcd. for C_17_H_19_ClN_3_O_3_, 348.1110; found, 348.1123.

*N-Hydroxy-4-{[(4-chlorophenylcarbamoylethyl)amino]methyl}benzamide* (**3l**). A khaki solid, 47 mg, yield: 66.0%; m.p. 161–163 °C; ^1^H-NMR: 2.46 (2H, t, *J* = 6.8, COCH_2_CH_2_), 2.76 (2H, t, *J* = 6.8, CH_2_CH_2_NH), 3.72 (2H, s, NHCH_2_), 7.34 (4H, m, Ar-H), 7.59 (2H, d, *J* = 8.8, Ar-H), 7.69 (2H, d, *J* = 8.0, Ar-H), 10.17 (1H, s, CONH); MS (ESI) *m/z*: 348 (M+H)^+^; HRMS (ESI) *m/z*: (M+H)^+^ calcd. for C_17_H_19_ClN_3_O_3_, 348.1109; found, 348.1102.

*N-Hydroxy-4-{[(4-methoxy-phenylcarbamoyl-ethyl)-amino]methyl}benzamide* (**3m**). A white solid, 13 mg, yield: 59.9%; m.p. 173–175 °C; ^1^H-NMR: 2.43 (2H, t, *J* = 6.4, COCH_2_CH_2_), 2.76 (2H, t, *J* = 6.4, CH_2_CH_2_NH), 3.71 (3H, s, OCH_3_), 3.75 (2H, s, NHCH_2_), 6.85 (2H, d, *J* = 9.2, Ar-H), 7.39 (2H, d, *J* = 8.0, Ar-H), 7.47 (2H, d, *J* = 9.2, Ar-H), 7.69 (2H, d, *J* = 8.0, Ar-H), 8.97 (1H, s, CONHOH), 9.87 (1H, s, CONH), 11.14 (1H, s, CONHOH); MS (ESI) *m/z*: 344 (M+H)^+^; HRMS (ESI) *m/z*: (M+H)^+^ calcd. for C_18_H_22_N_3_O_4_, 344.1605; found, 344.1594.

*N-Hydroxy-4-{[(3,4-dimethoxyphenylcarbamoylethyl)amino]methyl}benzamide* (**3n**). A white solid, 26 mg, yield: 44.3%; m.p. 175–176 °C; ^1^H-NMR: 2.72 (2H, t, *J* = 7.2, NHCH_2_CH_2_), 3.02 (2H, s, CH_2_CO), 3.31 (2H, t, *J* = 7.2, CH_2_CH_2_Ph), 3.62 (2H, s, NHCH_2_), 7.15–7.40 (7H, m, Ar-H), 7.68 (2H, d, *J* = 8.0, Ar-H), 7.84 (1H, m, NH), 8.97 (1H, s, CONHOH), 11.15 (1H, s, CONHOH); MS (ESI) *m/z*: 374 (M+H)^+^; HRMS (ESI) *m/z*: (M+H)^+^ calcd. for C_19_H_24_N_3_O_5_, 374.1711; found, 374.1713.

*N-Hydroxy-4-{[(4-methoxybenzylcarbamoylethyl)amino]methyl}benzamide* (**3o**). A khaki solid, 32 mg, yield: 52.9%; m.p. 152–154 °C; ^1^H-NMR: 2.28 (2H, t, *J* = 6.8, COCH_2_CH_2_), 2.69 (2H, t, *J* = 6.8, CH_2_CH_2_NH), 3.70 (2H, s, NHCH_2_), 3.71 (3H, s, OCH_3_), 4.17 (2H, d, CH_2_NHCO), 6.84 (2H, d, *J* = 8.8, Ar-H), 7.15 (2H, d, *J* = 8.8, Ar-H), 7.34 (2H, d, *J* = 8.0, Ar-H), 7.68 (2H, d, *J* = 8.0, Ar-H), 8.32 (1H, t, *J* = 5.6, CONH),8.96 (1H, s, CONHOH), 11.14 (1H, s, CONHOH); MS (ESI) *m/z*: 358 (M+H)^+^; HRMS (ESI) *m/z*: (M+H)^+^ calcd. for C_19_H_24_N_3_O_4_, 358.1761; found, 358.1771.

### 3.6. CLA-1 Up-Regulating Activity

CLA-1 up-regulating activity was analyzed as described previously [14]. Briefly, HepG2 cells stably transfected with pGL3-CLAP containing CLA-1 promoter region were seeded in 96-well plates at 5 × 10^4^ number/well in MEM (Hyclone, Logan, UT, USA) (100 μL) containing 10% FBS (Hyclone) and 600 μg/mL G418 (Invitrogen, Carlsbad, CA, USA). With ~80% confluence, the cells were washed once with PBS (pH 7.3, 137 mM NaCl, 2.7 mM KCl, 4.3 mM Na_2_HPO_4_, 1.4 mM KH_2_PO_4_), followed by incubation with MEM (200 μL) containing 0.1% vehicle (DMSO), positive control (2.5 μM SAHA or 3.0 μM TSA) or 10 μg/mL compounds. After 18 h incubation at 37 °C, cells were washed with PBS, and then the luciferase activity was detected using the Luciferase Assay System (Promega, Madison, WI, USA). EC_50_ values of the compounds were determined by a dose-response assay. Briefly, the above mentioned HepG2 cells were treated with the indicated concentrations of the compounds and detected by luciferase assay. The does-response curves were obtained and the apparent EC_50_ value for each compound was calculated using Sigma Plot 9.0.

### 3.7 Analysis for Cell Surface Expression by Flow Cytometry

Cell surface expression of CLA-1 was analyzed by flow cytometry as described previously [7]. Briefly, HepG2 cells were plated in 24-well dishes at 50,000 cells/well, followed by treatment for 24 h with 0.3 μM compounds or vehicle (0.1% DMSO). Then HepG2 cells were trypsinized from the plate, washed and resuspended in 4% paraform fixing solution, incubated overnight at 4 °C. After fixing, cells were blocked for 15 min at 4 °C in PBS containing 5% FBS. Cells were then incubated with monoclonal antibody to CLA-1 (BD Biosciences, San Jose, CA, USA) at a final dilution of 1:50 (4 °C, 1 h), followed by washing and staining with FITC-conjugated goat antibody to mouse IgG (1:100 dilution, 4 °C, 1 h). The cell suspension was centrifuged (800 × g, 3 min, 4 °C), the pellet was resuspended in PBS, and fluorescence intensity was analyzed using a BD FACSCalibur flow cytometer (BD Biosciences). 

### 3.8 Analysis of Cellular Uptake of DiI-labeled HDL by Flow Cytometry

For the cellular DiI-HDL uptake by HepG2 cells assays, the cell pretreatment was the same as for the cell surface expression assay. Cells were incubated with DiI-HDL (2 μg/mL) and 0.3 μM compounds or vehicle for 12 h at 37 °C, then washed with PBS and incubated with PBS containing 0.5% bovine serum albumin (BSA) and 2 mM EDTA for 1 h at 4 °C, detached from the plate by gentle pipetting. The cell suspension was centrifuged (3 min, 800 g, 4 °C), the obtained pellet was resuspended in PBS, and DiI fluorescence was analyzed using a BD FACSCalibur flow cytometer (BD Biosciences). 

## 4. Conclusions

A small library of hydroxamic acid compounds was constructed and the compounds were assessed for their up-regulating activity on CLA-1 expression in HepG2 cells. Among them, compound **1g** showed the best potency, with EC_50_ = 0.32 μM.
